# Measuring Electrolyte Impedance and Noise Simultaneously by Triangular Waveform Voltage and Principal Component Analysis

**DOI:** 10.3390/s16040576

**Published:** 2016-04-22

**Authors:** Shanzhi Xu, Peng Wang, Yonggui Dong

**Affiliations:** State Key Laboratory of Precision Measurement Technology and Instruments, Department of Precision Instrument, Tsinghua University, Beijing 100084, China; michaellegend@163.com (S.X.); dongyg@mail.tsinghua.edu.cn (Y.D.)

**Keywords:** solution impedance, planar-interdigitated electrode, conductivity, triangular waveform voltage, principal component analysis

## Abstract

In order to measure the impedance variation process in electrolyte solutions, a method of triangular waveform voltage excitation is investigated together with principal component analysis (PCA). Using triangular waveform voltage as the excitation signal, the response current during one duty cycle is sampled to construct a measurement vector. The measurement matrix is then constructed by the measurement vectors obtained from different measurements. After being processed by PCA, the changing information of solution impedance is contained in the loading vectors while the response current and noise information is contained in the score vectors. The measurement results of impedance variation by the proposed signal processing method are independent of the equivalent impedance model. The noise-induced problems encountered during equivalent impedance calculation are therefore avoided, and the real-time variation information of noise in the electrode-electrolyte interface can be extracted at the same time. Planar-interdigitated electrodes are experimentally tested for monitoring the KCl concentration variation process. Experimental results indicate that the measured impedance variation curve reflects the changing process of solution conductivity, and the amplitude distribution of the noise during one duty cycle can be utilized to analyze the contact conditions of the electrode and electrolyte interface.

## 1. Introduction

With the rapid development of impedance biosensors, the planar-interdigitated electrode (IDT) has been widely used in the research of solution impedance measurement, in applications such as water quality monitoring [1,2,3], pathogen concentration detection [4,5,6], and the identification of different substances by taste sensors [7,8]. Its wide use can be attributed to the advantages of small size, high sensitivity, and low sample volume requirement [9].

Sinusoidal waveform voltage excitation is commonly used in impedance measurement with IDT. In linear systems, the response current to a sinusoidal voltage excitation will be a standard sinusoidal signal at the same frequency. The amplitude and phase of the response current is then obtained to calculate the impedance under test. However, when measuring solution impedance, the nonlinear polarization of the electrode-electrolyte interface causes current waveform distortion to a certain degree, and therefore higher order harmonics will appear in the response current signals [10,11]. Especially at low frequencies (<1 kHz), polarization impedance is dominant and the nonlinear characteristics become more obvious [12]. Furthermore, the noise originating from the electrode interface, such as thermal noise, shot noise, and flicker noise (1/f noise) [13], also affects the quality of the response current signals. Above all, thermal noise and shot noise are white noise. Flicker noise is caused by the mass transfer process; the lower the frequency is, the more obvious the flicker noise becomes. In case of lower measuring frequencies (<1 kHz), a larger impedance with low phase will appear between the two IDT electrodes, and therefore the response current signals present with small amplitude with weak phase shift. So, the nonlinear polarization and interface noise seriously limit the accuracy of measurement results. Particularly, the noise contained in the response current signals increases the difficulty of extracting phase shift, thus severely impacting the accuracy of the impedance measurement.

Compared with sinusoidal waveform voltage excitation, triangular waveform voltage excitation is regarded as a more effective method for impedance measurement [14,15,16]. The solution resistance can be obtained from the linear scan region of triangular waveform voltage, while the capacitance can be obtained from the peak or valley points of triangular waveform voltage. This method can avoid the problem of large amplitude and phase errors due to low signal-to-noise ratio (SNR) of the response current signals. However, the equivalent circuit model is still needed to calculate equivalent impedance components and the noise-induced problems cannot be avoided. To the best of our knowledge, current research on triangular waveform voltage excitation is mainly working in a higher frequency region (>1 kHz). For example, the research group of Wu analyzed the influence of frequency on the measured resistance and capacitance of tap water by triangular waveform voltage excitation between 30 kHz and 50 kHz [14]. Then, the conductivity of KCl solutions and relative permittivity of molten salts were tested at the frequency of 50 kHz [14,15]. In biological impedance measurement, on the other hand, the impedance at low frequencies contains more information; e.g., molecule binding on the electrode surface [17] and the ion transport process of enzyme H^+^-ATPase in yeast respiration [18].

In addition, the noise in the response current signals is usually caused by the random fluctuations of ions from the electrode surface; therefore, noise signals contain useful information about the contact conditions in the electrode-electrolyte interface. For instance, the noise power spectrum can be used to provide the microcosmic variation information of the charge transfer resistance at the working electrode [19]. The electrode soaking process can be determined by the variation of the noise RMS amplitude [20]. Usually, the Fast Fourier Transform (FFT) is adopted to process the noise signals [21,22]. However, due to the average effect of the FFT algorithm, it is difficult to obtain real-time variation information of noise under the AC excitation signals.

In this paper, the impedance measurement method of triangular waveform voltage excitation at low frequencies is investigated. Using triangular waveform voltage as the excitation signal, the sampled response current is processed by principal component analysis (PCA) in order to avoid noise problems in the equivalent impedance calculation. At the same time, the real-time variation information of noise can be extracted to analyze the changing process at the electrode interface. Experimental results indicate that minor solution conductivity variation process and real-time noise variation process can be effectively measured simultaneously by the proposed method of triangular waveform voltage combined with principal component analysis.

## 2. Methods and Simulation Results

### 2.1. Impedance Measurement and Principal Component Analysis

**Figure 1:**
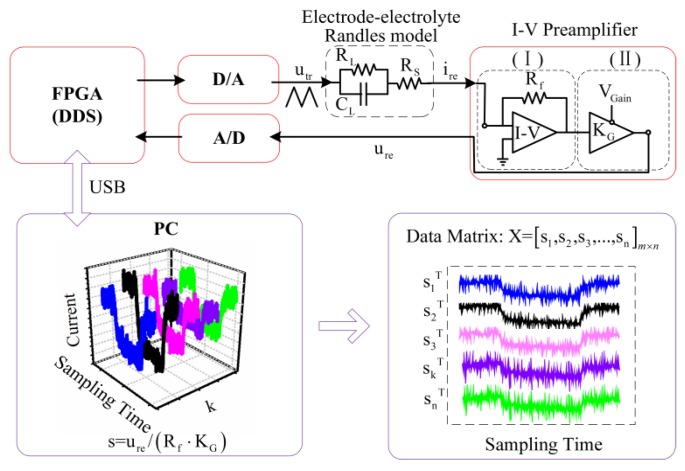
Schematic diagram of the impedance measurement system. FPGA: Field-programmable gate array; DDS: Direct digital synthesizer; D/A: Digital-to-analog converter; A/D: Analog-to-digital converter; (І): Current-to-voltage converter; (II): Voltage amplifier. PC: Personal computer.

The schematic diagram of the impedance measurement system is shown in Figure 1. The triangular waveform signal is digitally generated by a direct digital synthesizer (DDS) implemented in a field-programmable gate array (FPGA), and then transformed to analog triangular waveform voltage $u_{tr}$ by a digital-to-analog (D/A) converter. The response current $i_{re}$ of the IDT electrode under $u_{tr}$ excitation is amplified by a current-to-voltage converter (І) and a voltage amplifier (II). The output voltage $u_{re}$ is therefore determined by $u_{re}=i_{re}\cdot R_{f}\cdot K_{G}$. Finally, $u_{re}$ is sampled by an analog-to-digital (A/D) converter and transferred to a computer through a USB interface.

Since the triangular waveform voltage excitation is generated by a DDS module in FPGA, the signal sampling process can be controlled accurately. So, the response current during one duty cycle is sampled to construct a measurement vector $\vec{s}={\left(s_{1},s_{2},s_{3},\ldots ,s_{m}\right)}^{T}$, with *m* being the sample numbers. After successive measurements of the same time interval, n-component vectors are obtained as ${\vec{s}}_{k}={\left(s_{k,1},s_{k,2},s_{k,3},\ldots ,s_{k,m}\right)}^{T}$ (k=1,2,3,…,n). Hence, the data matrix is constructed for PCA processing, namely $X={\left[{\vec{s}}_{1},{\vec{s}}_{2},{\vec{s}}_{3},\ldots ,{\vec{s}}_{n}\right]}_{m\times n}$. After performing PCA on the covariance matrix of X, X is compressed into a reduced number of principal components (PCs):

(1) $${\hat{X}}_{r}=TP^{T}$$

The information of the response current waveform is contained in the score matrix T. The variation information of successive measurements is retained in the loading matrix P. The difference between the original data matrix and the reconstructed matrix by PCA is stored in the residual matrix $E=X-TP^{T}$, which reflects the noise information of the response current signals.

### 2.2. Simulation Results and Evaluation

**Figure 2:**
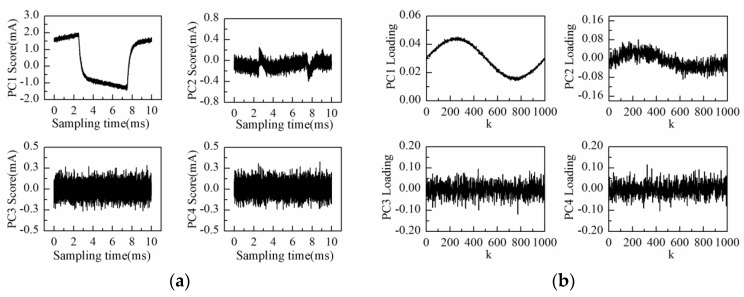
Results after principal component analysis (PCA) processing: (**a**) PC1–PC4 scores plot; (**b**) PC1–PC4 loadings plot.

**Figure 3:**
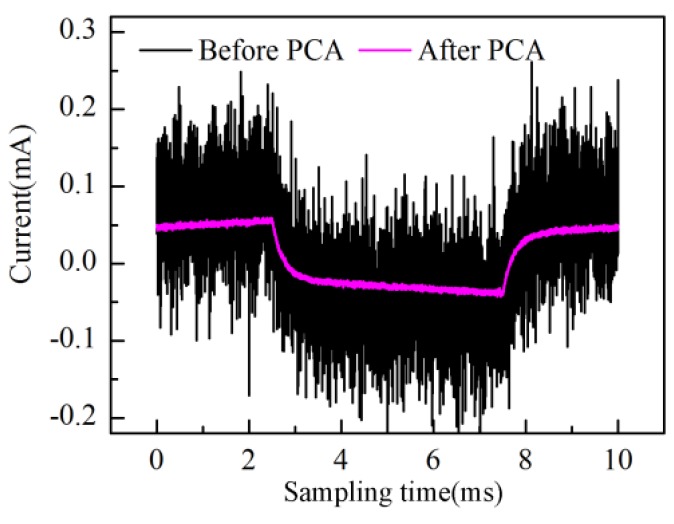
Current before and after PCA processing.

**Figure 4:**
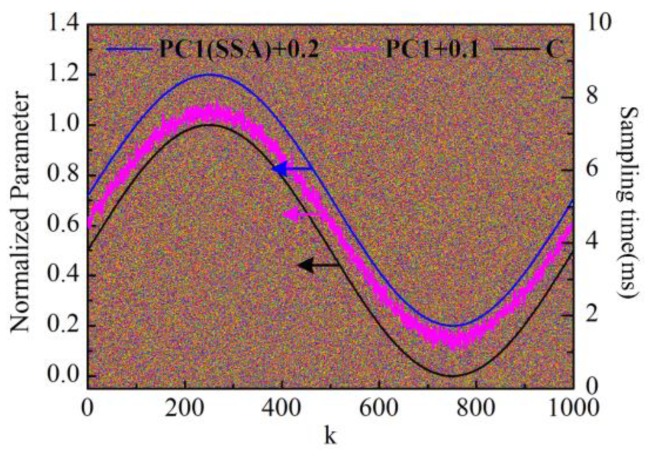
Comparison of the calculated results. SSA: Singular spectrum analysis. PC1(SSA) + 0.2: PC1 loading curve after SSA processing with a bias of 0.2; PC1 + 0.1: PC1 loading curve with a bias of 0.1; C: Capacitance variation curve.

Figure 2, Figure 3 and Figure 4 give numerical simulation results of the above measurement method. The equivalent circuit model under test is assumed to be a $R_{s}$ in series with a parallel combination of $R_{L}$ and $C_{L}$ as shown in Figure 1. The triangular waveform voltage excitation is set to be 100 Hz with 1 V peak-to-peak amplitude. White Gaussian noise with power spectral density (PSD) −85 dBW is added into the response current signals to simulate interfacial noise between electrode and electrolyte. The resistance values are set at a fixed value (*R_L_* = 50 kΩ, *R_s_* = 1 kΩ) while the capacitance varies sinusoidally (*C_L_* = 0.1{sin(0.002πk) + 2}*μF*, *k* = 0,1,2,…,1000), with k being the measurement time. After PCA processing, a scores and loadings plot of the first four principal components (PC1–PC4) is shown in Figure 2. As can be observed in Figure 2, the time-domain waveform information of response current is mostly contained in PC1 and PC2 scores; the PC1 score especially contains the main information of the response current waveform. On the other hand, the PC1 loading curve varies sinusoidally in consistence with capacitance. The PC2 loading curve contains large amounts of noise in comparison with the PC1 loading curve. The PC3 and PC4 loading curves reflect only random fluctuations. Therefore, PC1 describes the most important information regarding response current while PC2–PC4 mainly describe noise information. The response current of a certain measurement reconstructed by PC1 is illustrated in Figure 3. Compared with the original response current, the noise is effectively reduced after PCA processing and the abrupt current variation at the peak or valley points becomes more visible.

From Figure 2b, it can be seen that the PC1 loading curve ($p_{1}$) can considerably describe the impedance (capacitance) variation over time. In order to further filter out the fluctuations of $p_{1}$ and extract the impedance variation trend effectively, Singular Spectrum Analysis (SSA) [23] is proposed here to process the impedance variation curve of $p_{1}$. Firstly, the trajectory matrix (Hankle matrix) is constructed from $p_{1}$:

(2) $$Y=\left[Y_{1},Y_{2},\ldots ,Y_{K}\right]={\left(y_{ij}\right)}_{i,j=1}^{L,K}\;=\left(\begin{array}{cccc} p_{1} & p_{2} & \cdots & p_{K}\\ p_{2} & p_{3} & \cdots & p_{K+1}\\ \vdots & \vdots & \ddots & \vdots \\ p_{L} & p_{L+1} & \cdots & p_{k} \end{array}\right)$$

where L is the window length and K=k−L+1. Then, the trajectory matrix is reconstructed by the first *q* principal components after singular value decomposition (SVD):

(3) $$\hat{Y}=\sum_{i=1}^{q}\sqrt{\lambda_{i}}u_{i}v_{i}$$

Finally the smoothing impedance variation curve ${\hat{p}}_{1}$ is obtained by performing diagonal averaging on $\hat{Y}$, as shown in Figure 4. For comparison, impedance variation curve $p_{1}$ before SSA is also shown in the same figure together with the capacitance variation curve. For convenient observation, the values are normalized between 0 and 1. Meanwhile, PC1 loading curves before and after SSA processing are superimposed by the constant bias of 0.1 and 0.2, respectively. The calculated Pearson correlation coefficients of the PC1 loading curve before and after SSA processing are, respectively, 0.9980 and 0.9999 *versus* capacitance variation curve. Obviously, both can characterize the impedance (capacitance) variation well, and the result after being processed by SSA is even better. Furthermore, the residual noise signals are obtained by subtracting reconstructed signals by PC1 from the response current signals. For better observation of the noise variation, the amplitude values are normalized between 0 and 255 and then converted into a color image, as shown in Figure 4. The horizontal axis represents the noise variation with the measurement time k, and the vertical axis reflects how the noise in one sampling period varies with the amplitude of triangular waveform voltage. It can be seen that the amplitude level of the noise remains stable during the measurement in consistence with simulation settings.

## 3. Experimental Results and Discussion

### 3.1. Experimental Setup

**Figure 5:**
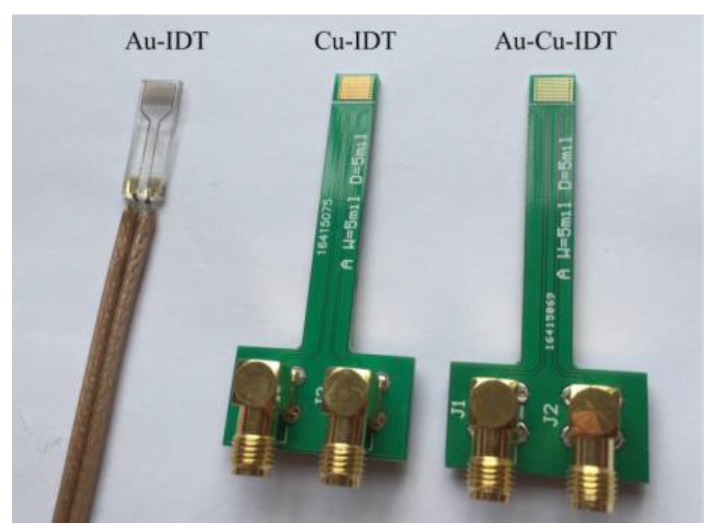
Diagram of three kinds of the planar-interdigitated electrodes (IDT) used. Au-IDT: Au electrode on Si substrate; Cu-IDT: Cu electrode on printed circuit board (PCB) substrate; and Au-Cu-IDT: Au electrode on PCB substrate.

The interdigitated electrodes immersed in the solutions had been tested experimentally by the measurement setup as shown in Figure 1. Three kinds of planar-interdigitated electrodes were used in the experiments; *i.e.*, Au electrode on Si substrate (Au-IDT), Cu electrode on PCB substrate (Cu-IDT) and Au electrode on PCB substrate (Au-Cu-IDT), as shown in Figure 5. All electrodes consist of eight interdigitated finger-pairs, with 5 mm finger length, 127 μm finger width, and 254 μm space between adjacent groups of interdigitated fingers. Au-IDT was fabricated on a silicon wafer, depositing a 20 nm layer of titanium (for enhancing Au adhesion) followed by a 200 nm layer of gold. Cu-IDT was fabricated on a printed circuit board (PCB) with a 35 μm layer of copper, and Au-Cu-IDT was fabricated by depositing a 30 nm layer of gold on Cu-IDT.

Au-IDT was reused in the experiment. Before each measurement, Au-IDT was cleaned in an ultrasonic bath using alcohol and then using deionized water for 10 min each. In order to avoid the electrode aging effect caused by Faradaic reactions of copper during the experiments, new electrodes were used in the Cu-IDT and Au-Cu-IDT experiments.

The sample solutions used in the experiments were KCl base solution (20 mmol/L), KCl background solution (50 mmol/L), thiol solution (10 mmol/L) and MPA solution (50 mmol/L). The thiol solution was prepared by dissolving 3-mercaptopropionic acid into anhydrous alcohol. The MPA solution was obtained by mixing 3-mercaptopropionic acid and the KCl background solution.

### 3.2. Experiment Results

In order to evaluate the influence of the triangular waveform voltage parameters, the impedance of the KCl base solution (20 mmol/L, 20 mL) was monitored using Au-IDT excited by triangular waveform voltage at different frequencies and amplitudes. The measurement peak-to-peak amplitudes chosen are 0.2 and 1 V, while the measurement frequencies are 10 Hz, 100 Hz, and 1 kHz.

**Figure 6:**
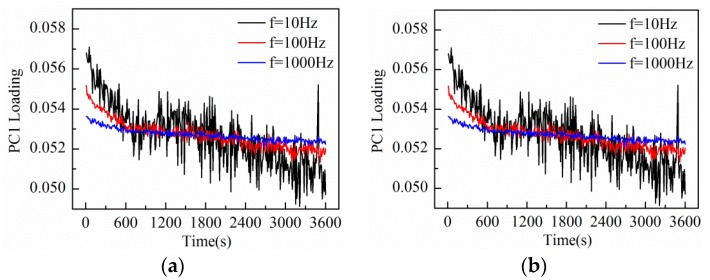
Measurement results of KCl base solution conducted with Au-IDT excited by triangular waveform voltage of different parameters: (**a**) A = 0.2 V; (**b**) A = 1 V.

The amplitude and frequency sweep test was conducted by the impedance measurement system at the interval of 10 s. At the same time, the response current during one duty cycle was sampled to construct the measurement matrix. After performing PCA on the data matrix of 1 h measurement results, the loadings plot of the first principal component (PC1) is shown in Figure 6. When the electrode was immersed in the KCl base solution, the equivalent impedance varied due to the charge distribution variation between electrode and electrolyte interface under external voltage excitation [24]. So, all the PC1 loading curves in Figure 6 show a general decrease trend in the initial period and then come to a steady state.

The experimental results indicate that the frequency and amplitude of the triangular waveform voltage both influence the measurement results. It can be seen that PC1 loading curves at higher frequencies are more relatively insensitive to the charge distribution variation of electrode interface, while PC1 loading curves at lower frequencies are more sensitive to the electrode interface variation. However, in the case of lower measurement frequencies, the response current signals present the characteristic of small amplitude with high noise level, thus leading to a poorer SNR. On the other hand, under the triangular waveform voltage excitation of larger amplitude, the SNR of the response current signal becomes better, so the measurement curves excited by 1 V amplitude is smoother than that excited by 0.2 V amplitude. In addition, we can see that PC1 loading curves excited by larger amplitude are more sensitive to the electrode interface variation. However, a voltage excitation amplitude that is too large will cause thermal effects at the electrode interface, even destroy the electrode. Consequently, considering the influencing factors of amplitude and frequency comprehensively, the triangular waveform voltage of 100 Hz frequency and 1 V peak-to-peak amplitude was chosen for the following measurement of solution impedance variation and real-time noise variation in electrode-electrolyte interface.

**Figure 7:**
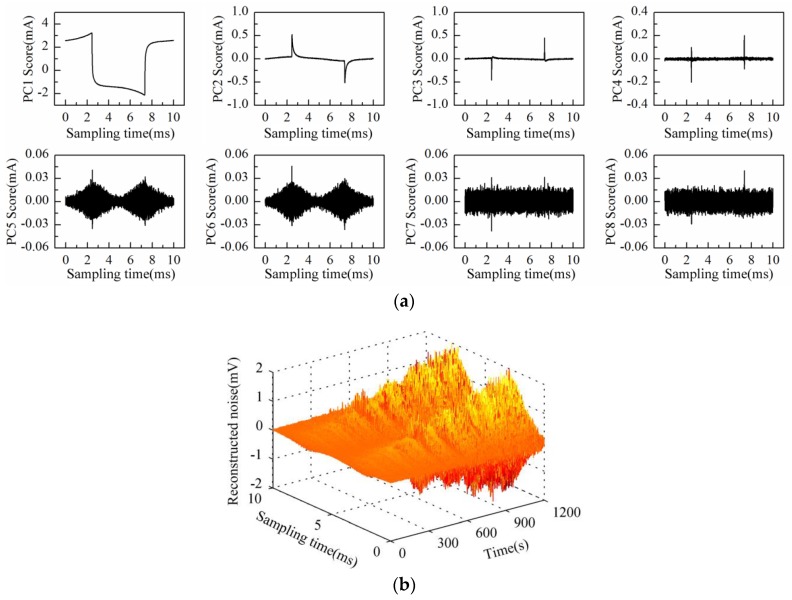
Measurement results of KCl solution conductivity variation process conducted with Au-IDT: (**a**) PC1–PC8 scores plot; (**b**) 3-D image of the reconstructed noise in one sampling period.

Then, the process of successive increase in solution concentration was monitored using Au-IDT. Low-concentration KCl solution, which is commonly used in biological impedance measurement, was adopted as the measuring object. By adding 1 mol/L KCl solution into the KCl base solution (20 mmol/L, 20 mL) every 1 min, the solution concentration was therefore increased linearly by 5 mmol/L after every addition. The response current was sampled by impedance measurement system at the interval of 1 s. Then, the data matrix was constructed with the obtained response current signals of 20 min measurement time. After performing PCA on the data matrix of measurement results, the scores plot of the first eight principal components (PC1–PC8) is shown in Figure 7a.

**Figure 8:**
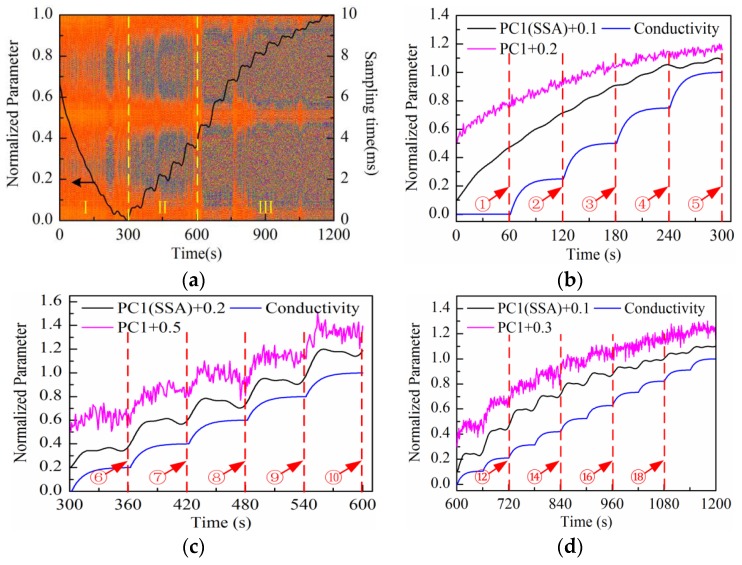
Measurement results of KCl solution conductivity variation process conducted with Au-IDT: (**a**) PC1 loading curve (after SSA) and the reconstructed noise image of the measurement period (0–20 min); (**b**) PC1 loading curve (before and after SSA) and theoretical conductivity variation curve of the first stage (0–5 min); (**c**) PC1 loading curve (before and after SSA) and theoretical conductivity variation curve of the second stage (5–10 min); (**d**) PC1 loading curve (before and after SSA) and theoretical conductivity variation curve of the third stage (10–20 min).

As can be observed in Figure 7a, the time-domain waveform information of response current in one sampling period is mostly contained in PC1 to PC4 scores; especially the PC1 score contains the main information of the response current waveform. On the other hand, the high frequency fluctuation information appears in the PC5 score and so on. It is notable that PC5 and PC6 scores present the “rhombus noise” related to voltage amplitude. In contrast, the noise in PC7 and PC8 scores is unrelated to voltage amplitude. In order to analyze the changing process of the “rhombus noise” with the concentration increase, the “rhombus noise” signals are reconstructed by the principal components of PC5 and PC6, as observed in Figure 7b (3-D image). It can be seen in Figure 7b that the noise amplitude shows a general trend of increase. The 3-D image is then converted to a 2-D image as shown in Figure 8a, in which the color variation of pixels stands for the distribution of noise amplitude.

The solid line in Figure 8a refers to the changing curve of PC1 loading after SSA and normalization processing. It is demonstrated that the PC1 loading curve presents different characteristics at different stages (І–II). In order to analyze the characteristics in more detail, the enlarged sectional curves at each stage are shown in Figure 8b–d. For comparison, the theoretical curve of solution conductivity variation caused by the addition of highly-concentrated solution and the PC1 loading curve before SSA are also presented in the same figure, with the arrows indicating the time of addition. Considering the diffusion process after every addition, the theoretical curve of solution conductivity variation is fitted by exponent rule. For convenient observation, the direction of the PC1 loading curve before and after SSA is reversed at the first stage (І). A certain constant bias value is superimposed to PC1 loading curves before and after SSA at each stage (see Figure 8b–d). It can be seen that the changing trend of solution conductivity can be extracted effectively by SSA processing. Especially during the measurement period after 720 s (adding at ⑫ in Figure 8d), the PC1 loading curve after SSA shows a more obvious stair-step increase feature compared to the curve before SSA.

At the first stage (see Figure 8b), the PC1 loading curve increases gradually so the solution impedance increases correspondingly. This is because the equivalent impedance varies due to the charge distribution variation of the electrode-electrolyte interface under the excitation of triangular waveform voltage at low frequencies. Therefore, it is hard to observe the impedance variation process caused by the addition of highly-concentrated solution. At the second stage (see Figure 8c), the PC1 loading curve shows the stair-step increase feature gradually, but is not stable because the curve maintains the descending trend as mentioned above. Compared with the first two stages, the curve shows a remarkable stair-step increase feature at the third stage (see Figure 8d). On the other hand, the amplitude distribution of “rhombus noise” varies regularly with the solution concentration increase. At the first stage, the noise level is low and unstable; meanwhile, the PC1 loading curve cannot effectively reflect the changing process of solution conductivity. At the second stage, the noise level becomes higher and the PC1 loading curve shows a gradual stair-step increase feature. At the third stage, the noise level remains stable and the PC1 loading curve shows a remarkable stair-step increase feature consistent with the theoretical conductivity variation curve.

Finally, cleaned Au-IDT was immersed in the thiol solution (10 mmol/L) for 4 h and then rinsed by deionized water. So, we obtained the modified Au-IDT covered with self-assembled monolayers (SAM-Au-IDT). The changing process of KCl solution conductivity had been monitored again using SAM-Au-IDT. The measurement process was the same as the experiment with Au-IDT shown in Figure 8.

**Figure 9:**
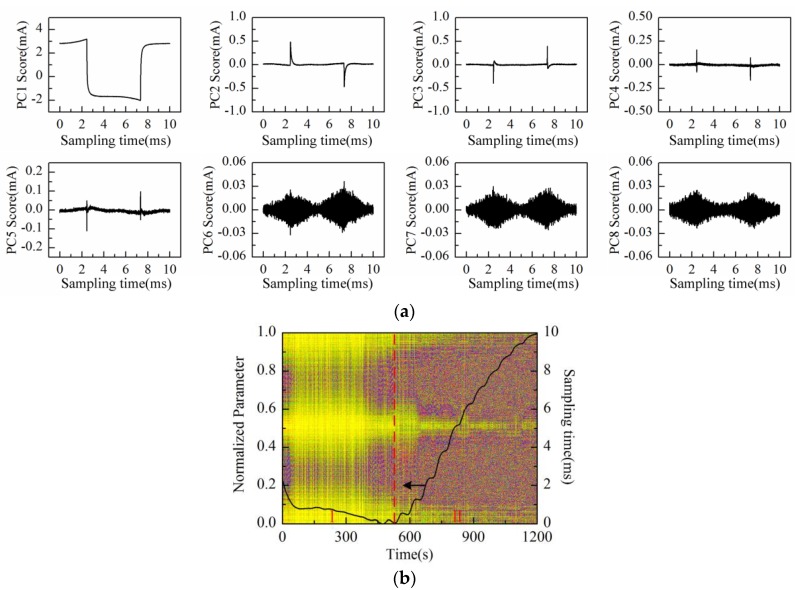
Measurement results of KCl solution conductivity variation conducted with the modified Au-IDT covered with self-assembled monolayers (SAM-Au-IDT): (**a**) PC1–PC8 scores plot; (**b**) PC1 loading curve (after SSA) and the reconstructed noise image of the measurement period (0–20 min).

The PC1 to PC8 scores plot after being processed by PCA is shown in Figure 9a. Obviously, the “rhombus noise” appears from PC6 to PC8. The noise image reconstructed by the above three principal components and the PC1 loading curve are shown in Figure 9b at the same time. It can be seen in Figure 9b that at the first stage (І), the PC1 loading curve of SAM-Au-IDT still shows a descending trend with significant fluctuations. However, the curve shows a remarkable stair-step increase feature at the second stage (II). Correspondingly, the noise amplitude in the first stage (I) is relatively unstable while it is increased gradually to a stable status at the second stage (II). Compared with Au-IDT, the SAM-Au-IDT curve has a relatively flatter slope but a longer descending time in the initial period. In addition, the smoothness of the SAM-Au-IDT curve is better in the stair-step increase period.

### 3.3. Discussion

In order to analyze the relationship between the “rhombus noise” and solution concentration, the KCl solution concentration of 50 mmol/L was selected as the measurement object corresponding to the appearance of obvious “rhombus noise” (360 s, adding at ⑥ in Figure 8a) during the measurement process. The experiment was then conducted using Au-IDT to investigate the noise characteristics of the selected KCl background solution (50 mmol/L, 20 mL). The measurement time was 1 h.

**Figure 10:**
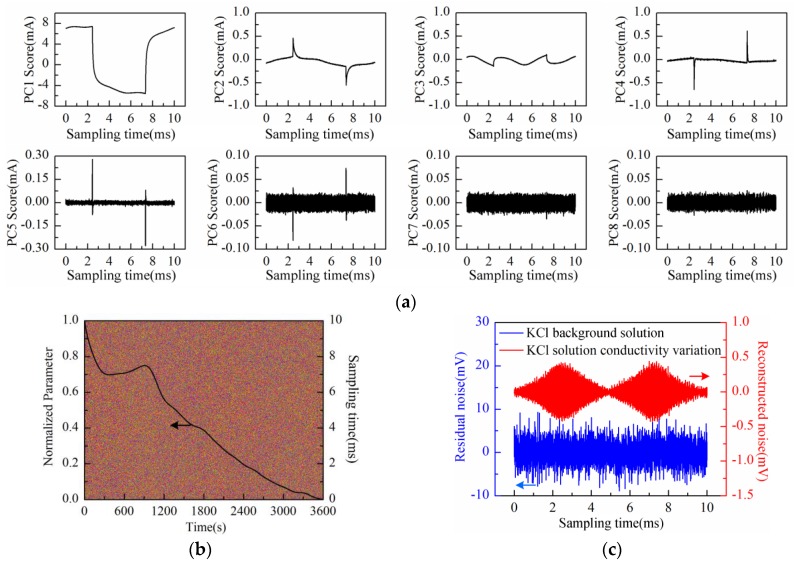
Measurement results of the KCl background solution conducted with Au-IDT: (**a**) PC1–PC8 scores plot; (**b**) PC1 loading curve (after SSA) and the residual noise image; (**c**) The residual noise signal at 360 s.

The scores plot of the first eight principal components (PC1–PC8) is illustrated in Figure 10a after performing PCA on the data matrix of measurement results. Obviously, PC1 to PC8 scores do not represent the “rhombus noise” related to voltage amplitude. PC1 to PC6 scores all contain partial information of the response current waveform. The residual noise, except the first six principal components, is therefore obtained as shown in Figure 10b (2-D image) together with the PC1 loading curve (after SSA and normalization processing).

As can be observed in Figure 10b, similar to the above experiment results, the PC1 loading curve appears in a general descending trend after immersing Au-IDT in the KCl background solution. However, the curve presents a slight upward fluctuation between 300 s and 1200 s. This phenomenon might be caused by the charge distribution variation of the electrode interface under the voltage excitation in highly-concentrated electrolyte solutions. According to the noise image, noise level remains relatively constant in the measurement process. The residual noise signal at 360 s is shown in Figure 10c. For comparison, the reconstructed noise signal at the same time in the experiment of Figure 8a is also illustrated in Figure 10c. From the above experimental results, it can be deduced that the “rhombus noise” has no direct connection with the absolute value of solution concentration.

According to the changing process of Figure 8a, another reason for the occurrence of the “rhombus noise” might be the dynamic process of solution concentration variation, thus causing the variation of contact conditions in the electrode-electrolyte interface. In order to verify this assumption, Au-IDT was soaked in aqueous MPA solution (50 mmol/L, 20 mL) for the impedance experiment. Since thiols bind to the gold surface rapidly to form self-assembled monolayers on the electrode interface [25], the contact status in the electrode-electrolyte interface will definitely be varied. The 50 mmol/L KCl background solution was used to prepare MPA solution, and the test process was the same as the above experiment (shown in Figure 10).

**Figure 11:**
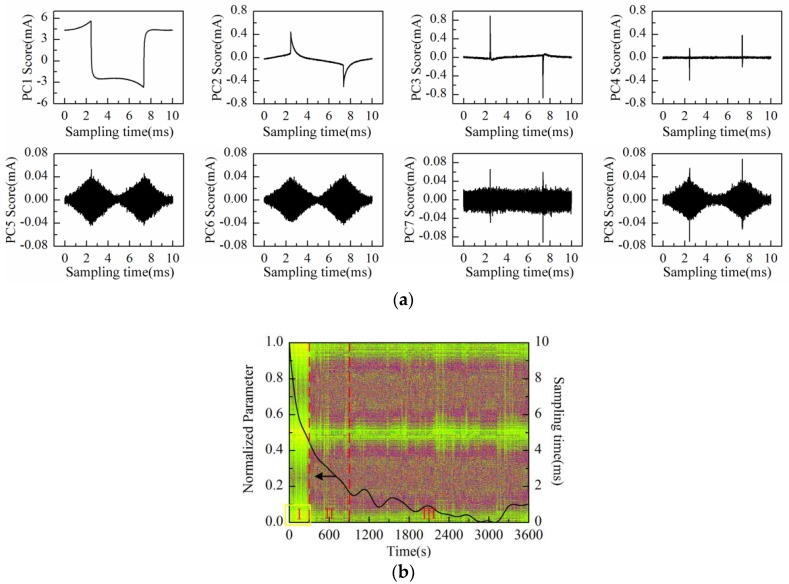
Measurement results of Mercaptopropionic Acid (MPA) solution conducted with Au-IDT: (**a**) PC1–PC8 scores plot; (**b**) PC1 loading curve (after SSA) and the reconstructed noise image.

The PC1–PC8 scores plot after being processed by PCA is shown in Figure 11a. Obviously, PC5, PC6, and PC8 represent the “rhombus noise” related to voltage amplitude. To extract the changing process of “rhombus noise”, the “rhombus noise” signals are reconstructed by the above-mentioned three principal components. The noise image is then obtained in Figure 11b, and the PC1 loading curve (after SSA and normalization processing) is also presented in the same figure. It can be seen in Figure 11b that the PC1 loading curve decreases gradually at the first stage (І) after soaking Au-IDT in MPA solution. With the formation of thiol monolayers on electrodes, the descending rate of the curve is slowed down at the second stage (II). After about 15 min (III), the descending trend stops and the curve shows the characteristic fluctuations. On the other hand, it can be seen from the noise image that the amplitude of “rhombus noise” is relatively small in the first stage (I). Then, the noise amplitude increases rapidly and comes to a steady state after about 5 min (II–III). Therefore, it could be inferred that the occurrence of the “rhombus noise” is related to the dynamic variation process of the electrode surface.

To further verify the above-mentioned assumption, Au-Cu-IDT was tested experimentally in the KCl background solution (50 mmol/L, 20 mL) instead. Since there was copper composition in Au-Cu-IDT, a Faradaic process caused by the dissolution of a small amount of copper during the measurement would also change the contact status of the electrode surface. The measurement time was also 1 h.

**Figure 12:**
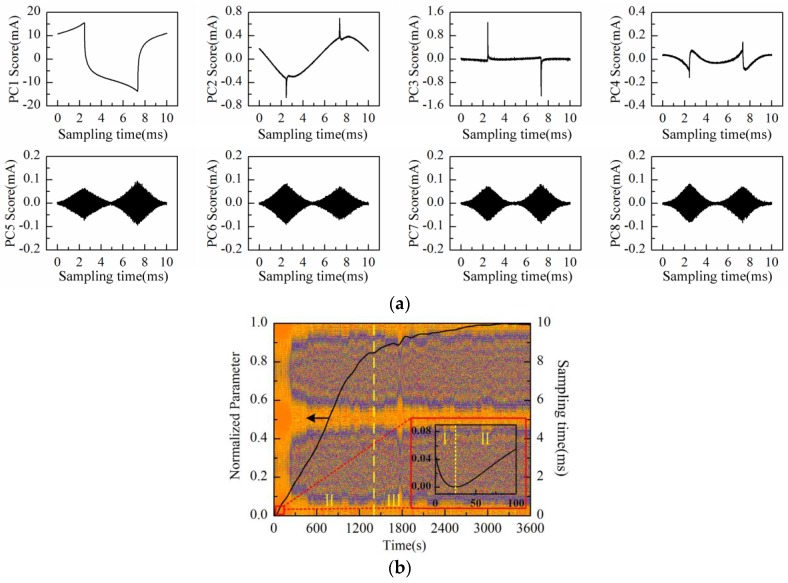
Measurement results of the KCl background solution conducted with Au-Cu-IDT: (**a**) PC1–PC8 scores plot; (**b**) PC1 loading curve (after SSA) and the reconstructed noise image.

The scores plot of the first eight principal components (PC1–PC8) is shown in Figure 12a. The “rhombus noise” appears from PC5 to PC8, in accordance with expectations. Reconstruction is performed by the above-mentioned four principal components. Figure 12b shows the noise image together with the PC1 loading curve (after SSA and normalization processing). The enlarged sectional view of the curve in the first 100 s is embedded in Figure 12b. As can be observed in Figure 12b, the noise level increases rapidly after about 300 s, which might be caused by the Faradaic reactions of the adhesive copper foil on the electrode surface. Similar to the measurement results of Au-IDT, the Au-Cu-IDT curve still shows a monotonic descending trend at the first stage (I). After about 20 s (II), the curve shows approximately the trend of linear increase. With the reaction process becoming stable gradually, the ascending rate of the curve is therefore slowed down after about 1400 s (Ш) and comes to a steady state gradually.

In order to further verify whether the occurrence of the “rhombus noise” was caused by Faradaic reactions on the electrode surface, Cu-IDT had been tested experimentally in the KCl background solution (50 mmol/L, 20 mL). The measurement time was also 1 h.

**Figure 13:**
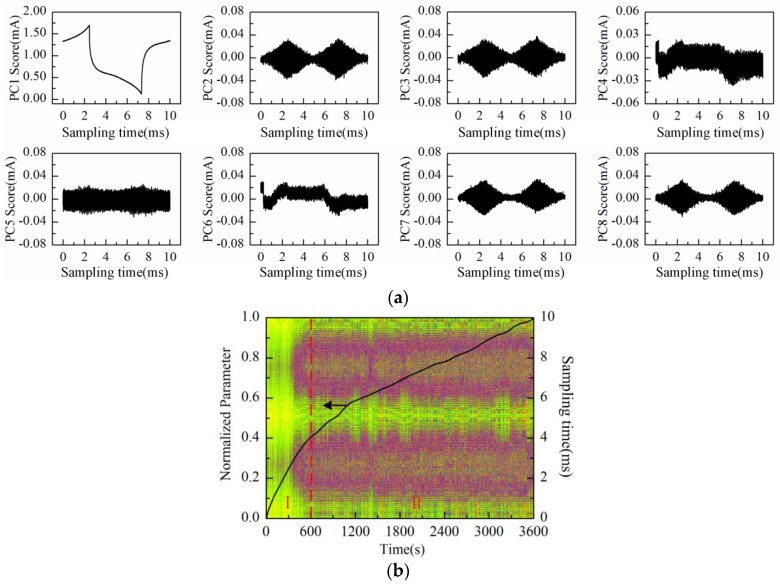
Measurement results of the KCl background solution conducted with Cu-IDT: (**a**) PC1–PC8 scores plot; (**b**) PC1 loading curve (after SSA) and the reconstructed noise image.

The PC1 to PC8 scores plot after being processed by PCA is shown in Figure 13a. Obviously, PC2, PC3, PC7, and PC8 scores represent the “rhombus noise”. Reconstruction is then performed by the above-mentioned four principal components. Figure 13b shows the noise image and PC1 loading curve (after SSA and normalization processing) simultaneously. After immersion of Cu-IDT in the KCl background solution (50 mmol/L, 20 mL), the Faradaic reactions are gradually enhanced. Therefore, the curve still approximately demonstrates the trend of linear increase at the first stage (I). Then, the Faradaic reactions are gradually weakened and stabilized at the second stage (II), and the ascending rate of the curve is gradually decreased. As can be observed from the noise image in Figure 13b, similar to the measurement results of Au-Cu-IDT, the noise level exhibits a relatively rapid rise after about 300 s, caused by the Faradaic reactions, which is consistent with the experimental results in [26,27].

The above-mentioned experimental verification concerning the occurrence of the “rhombus noise” is summarized in Table 1 below. Combined with all the measurement results, we can conclude that the changing process of the electrode–electrolyte contact interface will lead to the occurrence of such “rhombus noise”.

## 4. Conclusions

The impedance measurement method using triangular waveform voltage excitation at low frequencies is investigated. Planar-interdigitated electrodes are experimentally tested for monitoring the changing process of KCl solution conductivity. Further experimental verifications are performed for the occurrence of “rhombus noise” in the measurement results after being processed by PCA. The experimental results indicate that the method of triangular waveform voltage combined with principal component analysis can extract the minor conductivity variation process effectively. The “rhombus noise” signals after reconstruction can be utilized to determine the dynamic variation information of the electrode interface.

Compared with the traditional method of sinusoidal waveform voltage excitation, the proposed method can simultaneously extract the impedance variation information without equivalent circuit calculation and real-time noise variation information.

## Figures and Tables

**Table 1:** Experimental verification concerning the occurrence of the “rhombus noise”.

Verification Experiment	Occurrence of “Rhombus Noise”
KCl background solution conducted with Au-IDT	no
MPA solution conducted with Au-IDT	yes
KCl background solution conducted with Au-Cu-IDT	yes
KCl background solution conducted with Cu-IDT	yes
